# Supplementary material for: The Impact of Urinary Incontinence on Quality of Life: A Cross-Sectional Study in the Metropolitan City of Naples
Source: Geriatrics (Basel). 2020 Nov 20;5(4):96. doi: 10.3390/geriatrics5040096 (PMC7709681; doi:10.3390/geriatrics5040096)
Supplement: Supplementary file 1 [file geriatrics-05-00096-s001.zip › File 2 Questionnaire.docx]

**Quality of Life Questionnaire: Urinary incontinence**

Presentation

Sex: ☐ M ☐ F Age: _____ Occupation: _________________________________

Weight: ____________ Height: ________________ Body Mass Index (BMI): _________

Education: ☐ Primary;
 ☐ Secondary;
 ☐ High;
 ☐ Higher.

- Do you perform physical activity regularly? ☐ Yes ☐ No

- Which one of these pathologies you have?

☐ Arterial hypertension

☐ Urogenital prolapse

☐ Prostatic hypertrophy

☐ Diabetes

☐ Multiple sclerosis

☐ Parkinson disease

☐ Stroke

☐ Alzheimer disease

☐ Oncologic pathologies (e.g. bladder or prostate tumor)

☐ Urinary system infections

☐ Overactive bladder

☐ Spinal cord pathologies (e.g. herniated disk, injuries, syringomielia etc.)

☐ Constipation

☐ Kidney stones

☐ Urethra stenosis

☐ Spina bifida

☐ Hormonal alterations

- Sign eventual operations or other conditions that caused and hospitalization in the last years, indicating the date:

Date Operation / Hospitalization

__________________ _____________________________________________________

__________________ _____________________________________________________

- Do you take one of these drugs?

☐ Diuretics
☐ Laxatives

☐ Sedatives
☐ Muscle relaxants

☐ None of these

- Do you walk autonomously? ☐ Yes ☐ No

- For how much time have you been suffering of urinary incontinence?

☐ Few weeks
☐ Less than an year

☐ Just over 1 years
☐ Between 2 and 5 years

☐ More than 5 years

- How often do you use bathroom per day?

☐ Less than 6
☐ 6-10
☐ 10-15
☐ More than 15

- When, during the day, do you leak urine?

☐ Episodes during the day

☐ Episodes during the night

☐ Day and night

- How often do you leak urine?

☐ Rarely
☐ One or more times at week
☐ One time at day
☐ More than one time at day

- Select how much urine you lose every day, using as measure a size M absorbent (70 – 120 cm of hip and weight between 40 and 70 kg):


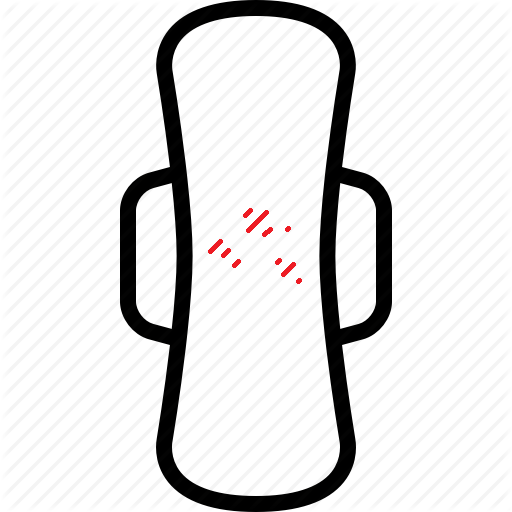

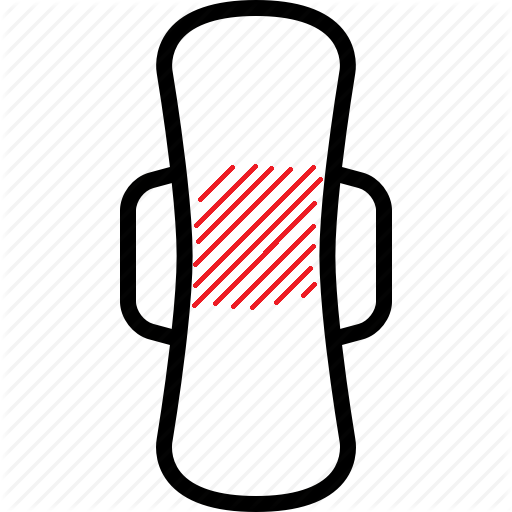

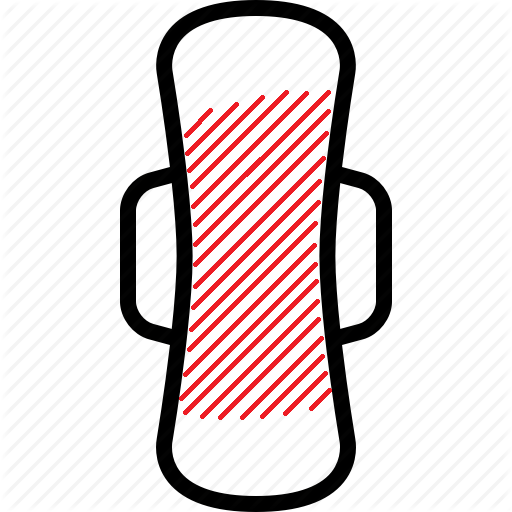

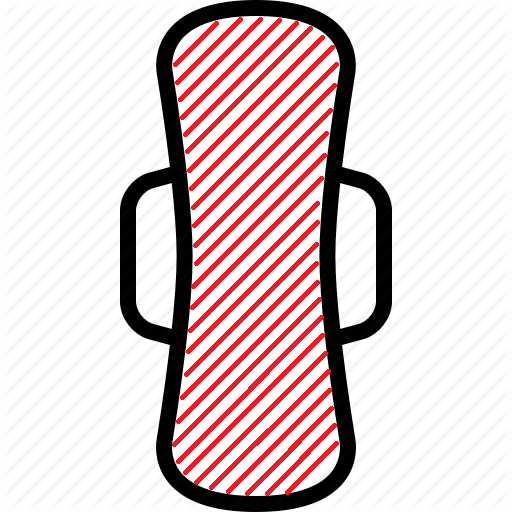


☐ ☐ ☐ ☐

- Have you ever felt like your bladder is not completely empty after used the toilet?
☐ Yes ☐ No ☐ Always

- Can you feel the stimulus of urination? ☐ Yes ☐ No

- When urination is started can you arrest it? ☐ Yes ☐ No

- In which of these circumstances do you leak urine? You can mark more than one answer.

☐ Before reaching the bathroom;
☐ When I cough or sneeze;
☐ During the sleep;
☐ During physical activity;
☐ Once dressed after urination;
☐ Without any particular reason

- Do you use one of these aids?
☐ None ☐ Traverse ☐ Absorbent ☐ Diaper ☐ Catheter

- How many changes of underwear/aids you make per day?

☐ 1 at day (24h) ☐ 2 at day ☐ 3 at day ☐ more than 3 at day

- Sign which of these treatments you have proved against urinary incontinence:

☐ Pelvic floor gym
☐ Anticholinergic drugs
☐ Topical estrogens
☐ Imipramine assumption
☐ Botulinum toxic
☐ Other
☐ None

- The treatments signed by you have improved you condition? ☐ Yes ☐ No

________________________________________________________________________________

Part dedicated to women

- Sign for each childbirth you had, if natural or cesarean, and specify if there have been difficulties:

|  |  | **Natural** | **Caesarean** |  | **Normal** | **Hard** |
| --- | --- | --- | --- | --- | --- | --- |
| ☐ I never gave birth |  | / / | / / |  | / / | / / |
| **Childbirth N° 1** |  | ☐ | ☐ |  | ☐ | ☐ |
| **Childbirth N° 2** |  | ☐ | ☐ |  | ☐ | ☐ |
| **Childbirth N° 3** |  | ☐ | ☐ |  | ☐ | ☐ |
| **Childbirth N° 4** |  | ☐ | ☐ |  | ☐ | ☐ |
| **Childbirth N° 5** |  | ☐ | ☐ |  | ☐ | ☐ |
| **Childbirth N° 6** |  | ☐ | ☐ |  | ☐ | ☐ |
| **Childbirth N° 7** |  | ☐ | ☐ |  | ☐ | ☐ |
| **Childbirth N° 8** |  | ☐ | ☐ |  | ☐ | ☐ |
| **Childbirth N° 9** |  | ☐ | ☐ |  | ☐ | ☐ |
| **Childbirth N° 10** |  | ☐ | ☐ |  | ☐ | ☐ |

Part dedicated to clinicians

Type of incontinence:

☐ Urgency urinary incontinence

☐ Stress-urinary incontinence

☐ Mixed urinary incontinence

☐ Overflow urinary incontinence

☐ Functional urinary incontinence

Severity:

| **Item** | **Score** | | | | | | | | | | | | | | |
| --- | --- | --- | --- | --- | --- | --- | --- | --- | --- | --- | --- | --- | --- | --- | --- |
| Deambulation autonomy | 0 |  |  |  |  |  |  |  |  |  | 5 |  |  |  |  |
| Urination frequency | 0 |  | 1 |  | 2 |  | 3 |  |  |  |  |  |  |  |  |
| When does he/she have leaks (day/night) |  |  | 1 |  | 2 |  |  |  |  |  |  |  |  |  |  |
| Leaks frequency |  |  | 1 |  | 2 |  | 3 |  | 4 |  |  |  |  |  |  |
| Quantity of urine he/she leaks |  |  | 1 |  | 2 |  | 3 |  | 4 |  |  |  |  |  |  |
| Sphincter control during urination | 0 |  | 1 |  |  |  |  |  |  |  |  |  |  |  |  |
| Circumstances in which he/she loses urine |  |  | 1 |  | 2 |  | 3 |  | 4 |  | 5 |  | 6 |  |  |
| Absorbent aid used | 0 |  | 1 |  | 2 |  | 3 |  |  |  |  |  |  |  |  |
| Changes made every day |  |  | 1 |  | 2 |  | 3 |  | 4 |  |  |  |  |  |  |

Total score: _____ / 32

**Quality of Life questionnaire**

|  | Not at all | A little | Moderately | Very much |
| --- | --- | --- | --- | --- |
| 1. Are you worried about wetting yourself because of leaks? | ☐ | ☐ | ☐ | ☐ |
| 1. Are you worried about sneezing or coughing because of your incontinence? | ☐ | ☐ | ☐ | ☐ |
| 1. When you visit new places, knowing the location of the bathroom does it worry you? | ☐ | ☐ | ☐ | ☐ |
| 1. Are you worried about not to reach on time to the bathroom? | ☐ | ☐ | ☐ | ☐ |
| 1. Are you worried about leaving home for long time? | ☐ | ☐ | ☐ | ☐ |
| 1. Are you worried about moving using public transport? | ☐ | ☐ | ☐ | ☐ |
| 1. Do you feel frustrated because incontinence doesn’t allow you to do what you want? | ☐ | ☐ | ☐ | ☐ |
| 1. Are you worried about the smell you may emit? | ☐ | ☐ | ☐ | ☐ |
| 1. Do you ever feel ashamed? | ☐ | ☐ | ☐ | ☐ |
| 1. Does your incontinence limit your clothes choices? | ☐ | ☐ | ☐ | ☐ |
| 1. Do you feel like you have no control on your life? | ☐ | ☐ | ☐ | ☐ |
| 1. Have you ever felt unable to enjoy the meetings with your friends? | ☐ | ☐ | ☐ | ☐ |
| 1. Does the incontinence interfere with your relation stability? | ☐ | ☐ | ☐ | ☐ |
| 1. Does the urinary incontinence limit your participation to social activities outdoor? | ☐ | ☐ | ☐ | ☐ |
| 1. Do you think your clinicians are disinterested about your health worries? | ☐ | ☐ | ☐ | ☐ |
| 1. Did urinary incontinence have a negative economic impact upon your life? | ☐ | ☐ | ☐ | ☐ |
| 1. Have you ever felt socially excluded? | ☐ | ☐ | ☐ | ☐ |
| 1. Does your health alter relations with your family? | ☐ | ☐ | ☐ | ☐ |
| 1. Do you feel like a weight to others? | ☐ | ☐ | ☐ | ☐ |
| 1. Do you feel discomfort to communicate with other? (talking, writing, listening, being understood and understanding) | ☐ | ☐ | ☐ | ☐ |
| 1. Do you feel discomfort to meet and to interact with new people? | ☐ | ☐ | ☐ | ☐ |
| 1. Does urinary incontinence interfere with your sexual desire or interest? | ☐ | ☐ | ☐ | ☐ |
|  | Not at all | A little | Moderately | Very much |
| 1. Do you consider yourself unsatisfied about your sexual relationship with your partner? | ☐ | ☐ | ☐ | ☐ |
| 1. Do thoughts about your health interfere with your sexual life? | ☐ | ☐ | ☐ | ☐ |
| 1. Does the urinary incontinence modify into negative your body image? | ☐ | ☐ | ☐ | ☐ |
| 1. Do the leaks interfere with your housework like cooking, ironing, washing etc.? | ☐ | ☐ | ☐ | ☐ |
| 1. Do the leaks interfere with your physical activities like jogging, swimming or working out? | ☐ | ☐ | ☐ | ☐ |
| 1. Do the leaks interfere with your hobbies like reading, watching movies, writing, painting etc.? | ☐ | ☐ | ☐ | ☐ |
| 1. Do the leaks interfere with your religious and/or spiritual activities? | ☐ | ☐ | ☐ | ☐ |
| 1. Have you ever felt discomfort on the work place? | ☐ | ☐ | ☐ | ☐ |
| 1. Does your health interfere with your work performance? | ☐ | ☐ | ☐ | ☐ |
| 1. Have you ever felt depressed? | ☐ | ☐ | ☐ | ☐ |
| 1. Have you ever felt oppressed by anxiety? | ☐ | ☐ | ☐ | ☐ |
| 1. Does the urinary incontinence force you to wake you up many times during the night? | ☐ | ☐ | ☐ | ☐ |
| 1. Do you pay attention to how much you drink when you are not home? | ☐ | ☐ | ☐ | ☐ |

Total score: _____ / 140
