# Supplementary material for: The Impact of Urinary Incontinence on Quality of Life: A Cross-Sectional Study in the Metropolitan City of Naples
Source: Geriatrics (Basel). 2020 Nov 20;5(4):96. doi: 10.3390/geriatrics5040096 (PMC7709681; doi:10.3390/geriatrics5040096)
Supplement: Supplementary file 1 [file geriatrics-05-00096-s001.zip › File 2 Questionnaire.pdf]

Quality of Life Questionnaire: Urinary incontinence

Presentation

Sex: ☐ M ☐ F Age: _____ Occupation: ________________________________

Weight: ____________ Height: ________________ Body Mass Index (BMI): _________

Education: ☐ Primary;

☐ Secondary;

☐ High;

☐ Higher.

- Do you perform physical activity regularly? ☐ Yes ☐ No

- Which one of these pathologies you have?

☐ Arterial hypertension

☐ Urogenital prolapse

☐ Prostatic hypertrophy

☐ Diabetes

☐ Multiple sclerosis

☐ Parkinson disease

☐ Stroke

☐ Alzheimer disease

☐ Oncologic pathologies (e.g. bladder or prostate tumor)

☐ Urinary system infections

☐ Overactive bladder

☐ Spinal cord pathologies (e.g. herniated disk, injuries, syringomielia etc.)

☐ Constipation

☐ Kidney stones

☐ Urethra stenosis

☐ Spina bifida

☐ Hormonal alterations

- Sign eventual operations or other conditions that caused and hospitalization in the last years, indicating the date:

Date	Operation / Hospitalization
__________________	____
__________________	____

- Do you take one of these drugs?

☐ Diuretics

☐ Laxatives

☐ Sedatives

☐ Muscle relaxants

☐ None of these

- Do you walk autonomously? ☐ Yes ☐ No

- For how much time have you been suffering of urinary incontinence?

☐ Few weeks ☐ Just over 1 years ☐ More than 5 years

☐ Less than an year ☐ Between 2 and 5 years

- How often do you use bathroom per day?

☐ Less than 6 ☐ 10-15

☐ 6-10 ☐ More than 15

- When, during the day, do you leak urine?

☐ Episodes during the day ☐ Episodes during the night ☐ Day and night

- How often do you leak urine?

☐ Rarely ☐ One time at day

☐ One or more times at week ☐ More than one time at day

- Select how much urine you lose every day, using as measure a size M absorbent (70 – 120 cm of hip and weight between 40 and 70 kg):

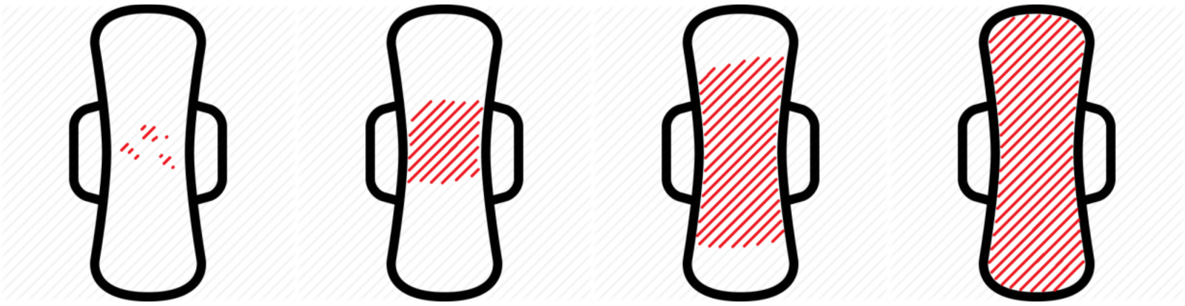

☐ ☐ ☐ ☐

- Have you ever felt like your bladder is not completely empty after used the toilet?

☐ Yes ☐ No ☐ Always

- Can you feel the stimulus of urination? ☐ Yes ☐ No

- When urination is started can you arrest it? ☐ Yes ☐ No

- In which of these circumstances do you leak urine? You can mark more than one answer.

☐ Before reaching the bathroom;

☐ When I cough or sneeze;

☐ During the sleep;

☐ During physical activity;

☐ Once dressed after urination;

☐ Without any particular reason

- Do you use one of these aids?

☐ None ☐ Traverse ☐ Absorbent ☐ Diaper ☐ Catheter

- How many changes of underwear/aids you make per day?

☐ 1 at day (24h) ☐ 2 at day ☐ 3 at day ☐ more than 3 at day

- Sign which of these treatments you have proved against urinary incontinence:

☐ Pelvic floor gym

☐ Anticholinergic drugs

☐ Topical estrogens

☐ Imipramine assumption

☐ Botulinum toxic

☐ Other

☐ None

- The treatments signed by you have improved you condition? ☐ Yes ☐ No

---

Part dedicated to women

- Sign for each childbirth you had, if natural or cesarean, and specify if there have been difficulties:

	**Natural**	**Caesarean**		**Normal**	**Hard**
☐ I never gave birth	/ /	/ /		/ /	/ /
**Childbirth N° 1**	☐	☐		☐	☐
**Childbirth N° 2**	☐	☐		☐	☐
**Childbirth N° 3**	☐	☐		☐	☐
**Childbirth N° 4**	☐	☐		☐	☐
**Childbirth N° 5**	☐	☐		☐	☐
**Childbirth N° 6**	☐	☐		☐	☐
**Childbirth N° 7**	☐	☐		☐	☐
**Childbirth N° 8**	☐	☐		☐	☐
**Childbirth N° 9**	☐	☐		☐	☐
**Childbirth N° 10**	☐	☐		☐	☐

Part dedicated to clinicians

Type of incontinence:

☐ Urgency urinary incontinence

☐ Stress-urinary incontinence

☐ Mixed urinary incontinence

☐ Overflow urinary incontinence

☐ Functional urinary incontinence

Severity:

Item	Score						
Deambulation autonomy	0					5	
Urination frequency	0	1	2	3			
When does he/she have leaks (day/night)		1	2				
Leaks frequency		1	2	3	4		
Quantity of urine he/she leaks		1	2	3	4		
Sphincter control during urination	0	1					
Circumstances in which he/she loses urine		1	2	3	4	5	6
Absorbent aid used	0	1	2	3			
Changes made every day		1	2	3	4		

Total score: _____ / 32

Quality of Life questionnaire

	Not at all	A little	Moderately	Very much
1. Are you worried about wetting yourself because of leaks?	☐	☐	☐	☐
2. Are you worried about sneezing or coughing because of your incontinence?	☐	☐	☐	☐
3. When you visit new places, knowing the location of the bathroom does it worry you?	☐	☐	☐	☐
4. Are you worried about not to reach on time to the bathroom?	☐	☐	☐	☐
5. Are you worried about leaving home for long time?	☐	☐	☐	☐
6. Are you worried about moving using public transport?	☐	☐	☐	☐
7. Do you feel frustrated because incontinence doesn't allow you to do what you want?	☐	☐	☐	☐
8. Are you worried about the smell you may emit?	☐	☐	☐	☐
9. Do you ever feel ashamed?	☐	☐	☐	☐
10. Does your incontinence limit your clothes choices?	☐	☐	☐	☐
11. Do you feel like you have no control on your life?	☐	☐	☐	☐
12. Have you ever felt unable to enjoy the meetings with your friends?	☐	☐	☐	☐
13. Does the incontinence interfere with your relation stability?	☐	☐	☐	☐
14. Does the urinary incontinence limit your participation to social activities outdoor?	☐	☐	☐	☐
15. Do you think your clinicians are disinterested about your health worries?	☐	☐	☐	☐
16. Did urinary incontinence have a negative economic impact upon your life?	☐	☐	☐	☐
17. Have you ever felt socially excluded?	☐	☐	☐	☐
18. Does your health alter relations with your family?	☐	☐	☐	☐
19. Do you feel like a weight to others?	☐	☐	☐	☐
20. Do you feel discomfort to communicate with other? (talking, writing, listening, being understood and understanding)	☐	☐	☐	☐
21. Do you feel discomfort to meet and to interact with new people?	☐	☐	☐	☐
22. Does urinary incontinence interfere with your sexual desire or interest?	☐	☐	☐	☐

	Not at all	A little	Moderately	Very much
23. Do you consider yourself unsatisfied about your sexual relationship with your partner?	☐	☐	☐	☐
24. Do thoughts about your health interfere with your sexual life?	☐	☐	☐	☐
25. Does the urinary incontinence modify into negative your body image?	☐	☐	☐	☐
26. Do the leaks interfere with your housework like cooking, ironing, washing etc.?	☐	☐	☐	☐
27. Do the leaks interfere with your physical activities like jogging, swimming or working out?	☐	☐	☐	☐
28. Do the leaks interfere with your hobbies like reading, watching movies, writing, painting etc.?	☐	☐	☐	☐
29. Do the leaks interfere with your religious and/or spiritual activities?	☐	☐	☐	☐
30. Have you ever felt discomfort on the work place?	☐	☐	☐	☐
31. Does your health interfere with your work performance?	☐	☐	☐	☐
32. Have you ever felt depressed?	☐	☐	☐	☐
33. Have you ever felt oppressed by anxiety?	☐	☐	☐	☐
34. Does the urinary incontinence force you to wake you up many times during the night?	☐	☐	☐	☐
35. Do you pay attention to how much you drink when you are not home?	☐	☐	☐	☐

Total score: _____ / 140
