# Supplementary material for: The Impact of Urinary Incontinence on Quality of Life: A Cross-Sectional Study in the Metropolitan City of Naples
Source: Geriatrics (Basel). 2020 Nov 20;5(4):96. doi: 10.3390/geriatrics5040096 (PMC7709681; doi:10.3390/geriatrics5040096)
Supplement: Supplementary file 1 [file geriatrics-05-00096-s001.zip › File 1 Informed consent.docx]

**Informed consent under the EU 2016/679 Regulation (GDPR) for Personal Data Protection**

In compliance with the fulfillments required by the art. 13 and 14 of EU 2016/679 Regulation for personal data protection we communicate to you the modalities used to collect and process your data:

1. General, personal and, where required “particular”, data are collected exclusively for the research Study of a Master’s Degree Thesis in “Science rehabilitation” at the University of Naples Federico II and its eventual publication. The research study will regard “Quality of life”.
2. Data are collected with the use of an anonymous papery survey. Data will be read and processed by authorised personal in a very responsible way.
3. Your personal data won’t be shed, but they will be eventually communicated to other organisms which collaborate in the study realisation.
4. Data will be stored up and processed anonymously until the discussion of the graduation thesis, and in any case, they won’t be stored up for more than a year. Moreover, they won’t be shed to extra-European Union countries.
5. During the processing of your personal data, we will consider your rights (articled from 15 to 22 of the EU 2016/679 Regulation), such as the right of access, the rectification or deletion (right to be forgotten), the restriction of processing, the right to object and the right to propose a complaint to the Privacy Protection Supervisor. The requests to apply one of your rights must be sent to the processor, using the below address.
6. The data processor and controller is the Doc. Benedetto Giardulli
    Telephone: +39 3313532560 Email: [benedettogiardulli@gmail.com](mailto:benedettogiardulli@gmail.com)

The undersigned ____________________________________ gives consent, pursuant to the art. 7 of GDPR, for the processing of personal data for the above-mentioned finalities.

Place and Date: _______________________ Signature: ________________________________
