# Supplementary material for: The Impact of Urinary Incontinence on Quality of Life: A Cross-Sectional Study in the Metropolitan City of Naples
Source: Geriatrics (Basel). 2020 Nov 20;5(4):96. doi: 10.3390/geriatrics5040096 (PMC7709681; doi:10.3390/geriatrics5040096)
Supplement: Supplementary file 1 [file geriatrics-05-00096-s001.zip › File 3 STROBE checklist.doc]

STROBE Statement—checklist of items that should be included in reports of observational studies

|  | Item No | Recommendation |
| --- | --- | --- |
| **Title and abstract** | 1 | The impact of urinary incontinence on quality of life: a cross-sectional study in the Province of Naples, Italy |
|  |  | More than 200 million people worldwide experience urinary incontinence (UI), especially women and elderly, which imply hygienic and psychosocial problems. To test the impact that UI has on quality of life we built a multidimensional specific questionnaire that we gave to 25 residents of the Province of Naples, Italy, to analyse the results. The secondary objective of this study was to find which variables affect QOL and symptom severity in these patients.  Most of the participants had a mild impairment (60%) concerning social life and self-perception, especially who had an education superior to the “Primary” (p=.036). Who followed a pelvic floor rehabilitation program resulted with a higher quality of life impairment (p=.002). Overflow urinary incontinence was associated with a higher deterioration of the aspirational and occupational domain (p=.044). Symptoms severity were, instead, worse in who had comorbidities (p=.038), in who had a high Body Mass Index (p=.008), and in who used diuretics (p=.007).  We concluded that urinary incontinence affects physical, psychological and social spheres of the patients, especially in who have a high education and who follows a pelvic rehabilitation program. Further studies are needed to extend the sample and study the sensibility of the proposed test. |
| Introduction | | |
| Background/rationale | 2 | Urinary incontinence is a relevant hygienic and psychosocial problem, characterised by the involuntary loss of urine that influences many aspects of patients’ life such as family, occupation and even sex, and therefore it may decrease patients’ quality of life. |
| Objectives | 3 | Primary: to evaluate the impact of urinary incontinence on quality of life among residents living in Province of Naples, Italy.  Secondary: to find which variables affect quality of life and symptoms severity in these patients. |
| Methods | | |
| Study design | 4 | Multicentre cross-sectional study. |
| Setting | 5 | “Ufficio Prescrizione Ausili Assorbenti” at District 44 of the “Azienda Sanitaria Locale Napoli 2 Nord”; “Reparto di Urologia” and “Ambulatorio di Urodinamica” at “Azienda Ospedaliera di Rilievo Nazionale A. Cardarelli”. |
| Participants | 6 | *Cross-sectional study*—Patients were eligible if: they had urinary incontinence diagnosed; their age was superior to 18 years; time from the onset of UI was superior to 1 year; they were under treatment by the Italian National Health System. Patients were excluded if they had cognitive impairments that would have compromised the understanding of the study or if their questionnaire was incomplete. The recruitment was voluntary. |
| Variables | 7 | Outcomes: quality of life impairment score (three different domains); severity score.  Predictors: age; sex (male or female); education (primary or higher); body mass index; comorbidity; delivery numbers; pelvic floor rehabilitation; types of incontinence; time from symptoms onset; daily changes (24h); bathroom uses per day; use of aids; use of diuretics; use of sedatives. |
| Data sources/ measurement | 8* | Quality of life (and domains) impairment and symptoms severity were assessed with an original questionnaire (QOL-UI).  All the other variables information are extracted from the same questionnaire. |
| Bias | 9 | Selection bias; small sample; reliability and validity of the tool proposed. |
| Study size | 10 | A sample composed of 28 patients, of which 3 excluded. |
| Quantitative variables | 11 | For the quantitative variables were measured means and standard deviations. In addition, the Pearson correlation coefficient (*r*) and determination coefficient (*r*^2^) in the linear regression graph were calculated in order to analyse correlations between quantitative variables.  The sample has been stratified based on different category variables, in order to make a comparison between the medians of two or more independent groups. |
| Statistical methods | 12 | (*a*) The analysis studied ordinal and continuous variables, characterised, respectively, by numbers and percentages, and by means and standard deviations. To study comorbidity, the absolute frequency and the relative one in percentage were calculated |
|  |  | (*b*) Nonparametric tests, like Kruskal–Wallis and Mann-Whitney with a p-value threshold of 0.05, were used to examine subgroups correlations. |
|  |  | (*c*) If the questionnaire presented missing data than it was excluded by the study and analysis. |

| Results | | |
| --- | --- | --- |
| Participants | 13* | 28 patients were recruited and assessed with QOL-UI, all older than 18 years. Three participants were excluded because they did not fill the questionnaire correctly. No exclusion due to cognitive impairments was made.  The patients included in the study were 25. |
| Descriptive data | 14* | The sample had a majority of women (60%), and about the 84% of the participants were above 50 years old. Of the participants, 28% had received an education above the primary level, 16% declared that they were employed, and 80% had a BMI greater than the Regular cut-off point (25.0). The type of incontinence most represented was the mixed one (95%). The main causes of UI were, for women, urogenital prolapse (44%), and for men, prostatic hypertrophy (16%). Comorbidity was common in the sample, and the most frequent condition was hypertension (56%), followed by diabetes (40%). |
| Outcome data | 15* | The numerical outcomes calculated from patients’ questionnaires were: total quality of life score; being score; belonging score; becoming score; severity score; age, body mass index. |
| Main results | 16 | (*a*) the analysed sample, for the most, presented a moderate severity of the symptoms (mean 17.6; DS 4.36), and a QOL total score indicating moderate impairment (mean 91.24; DS 20.11), but the influence of severity on QOL has not been significantly appreciated (r = 0.30). |
|  |  | (*b*) Severity was affected by age (r = 0.59, P = .001) and BMI (r = 0.58, P = .002) according to the Pearson correlation analysis, and by comorbidities (U = 24, P = .038), absorbent aids use (U = 22.5, P = .030), diuretic use (U = 24, P = .007), and BMI again (K = 9.47, P = .008) according to nonparametric tests.  QOL total score, according to nonparametric tests, was affected by the educational level (U = 28, P = .036) and pelvic floor rehabilitation (U = 8, P = .002). No positive significant correlation was found between QOL total score and either sex or age (r = 0.13), nor for age among women (r = 0.02). |
| Other analyses | 17 | Analysing the QOL domains, we found that patients presented, for the most part, less impairment in the Belonging domain (slight in the 52% of the sample), while the Being and Becoming domains were more compromised (mild impairment in 56% and 52% of participants, respectively). Using nonparametric tests on QOL domains, we also found that the Being domain was more compromised in those who had a low educational level (U = 24, P = .026); the Becoming domain was more compromised in men (U = 39, P = .048) and in overflow UI type (K = 6.20, P = .044); all domains were more compromised in those who followed a pelvic floor rehabilitation program (U = 11.5, P = .009; U = 8.5, P = .002; U = 9, P = .002; Being, Belonging and Becoming, respectively). |
| Discussion | | |
| Key results | 18 | UI is cause of embarrassment because it can interfere with self-image and consequently also self-esteem and attractiveness (Aylaz R. et al., 2016). The Being domain, which includes safety and self-concept, was moderately compromised in 73.3% of women, and in 20% of cases severely compromised. The same domain deterioration was observed in men. Low self-esteem and a negative body image are also associated with a low level of education (Kurt E. et al., 2013). Indeed, in the study there was a sensitive impairment of the Being domain in those whose highest educational level completed was primary school.  BMI is strongly correlated with the severity of UI (Yan W. et al., 2018), but there was no greater impairment of QOL in subjects with higher BMI.  Absorbent aids play a fundamental role in changing the body image and self-esteem of subjects with UI (Gümüșsoy S. et al., 2019; Nitti V. W. et al., 2014; Getliffe K. et al., 2007). Indeed, there is a compromise in the Becoming domain because self-determination is lacking in these subjects. In some cases, even the Belonging domain is compromised, especially when subjects sacrifice their social life for fear that absorbent aids may give off bad smells. Instead, the participants who claimed to change more than three aids a day had a more compromised domain of Being (Barentesen J.A. et al., 2012).  Sexual satisfaction and intimate relationships did not show significant impairment in the study, although many studies have shown how much UI affects the sexual sphere (Moore C.K., 2010, 299-303).  Those who followed a pelvic floor rehabilitation program had a significantly greater impact on QOL in all domains, even though it is a valid tool able to improve UI symptoms (Dumoulin C. et al., 2018). |
| Limitations | 19 | The study presents a favourable sampling that includes only subjects who voluntarily chose to participate in the study, and therefore there is a selection bias. Furthermore, the reliability and validity of the tool used would deserve further investigation. At last, the study was carried out on a small sample (<30); therefore, it would be interesting to extend the sample size to observe the extension of the results. |
| Interpretation | 20 | Urinary incontinence can indirectly affect the physical, psychological and social spheres of both men and women because it may significantly interfere with patients’ family and social life. Symptoms should be carefully evaluated from a holistic and multiperspective point of view. Any therapeutic program should be personalised to match the patient’s individual needs. |
| Generalisability | 21 | More studies are needed to generalise the study results to a wider sample. |
| Other information | | |
| Funding | 22 | No funding. |
